# Pandemic policies and breastfeeding: A cross-sectional study during the onset of COVID-19 in the United States

**DOI:** 10.3389/fsoc.2022.958108

**Published:** 2022-11-03

**Authors:** Aunchalee E. L. Palmquist, Cecília Tomori, Katherine Tumlinson, Carolyn Fox, Stephanie Chung, E. A. Quinn

**Affiliations:** ^1^Department of Maternal and Child Health, Gillings School of Global Public Health, University of North Carolina at Chapel Hill, Chapel Hill, NC, United States; ^2^Carolina Population Center, University of North Carolina at Chapel Hill, Chapel Hill, NC, United States; ^3^Johns Hopkins School of Nursing, Johns Hopkins University, Baltimore, MD, United States; ^4^Johns Hopkins Bloomberg School of Public Health, Johns Hopkins University, Baltimore, MD, United States; ^5^Department of Anthropology, Washington University in St. Louis, St. Louis, MO, United States

**Keywords:** breastfeeding, lactation, infant feeding, COVID-19, policy, paid leave, health equity

## Abstract

The United States is one of the few countries, and the only high-income country, that does not federally mandate protection of postpartum employment through paid postpartum maternity and family leave policies. At the onset of the COVID-19 pandemic in the U.S., stay-at-home orders were implemented nationally, creating a natural experiment in which to document the effects of *de facto* paid leave on infant feeding practices in the first postpartum year. The purpose of this cross-sectional, mixed-methods study was to describe infant and young child feeding intentions, practices, decision-making, and experiences during the first wave of the COVID-19 pandemic in the U.S. Quantitative and qualitative data were collected March 27–May 31, 2020 via online survey among a convenience sample of respondents, ages 18 years and older, who were currently feeding a child 2 years of age or younger, yielding 1,437 eligible responses. Nearly all (97%) respondents indicated an intention to feed their infant exclusively with human milk in the first 6 months. A majority of respondents who were breastfeeding (66%) reported no change in breastfeeding frequency after the implementation of COVID-19 stay-at-home orders. However, thirty-one percent indicated that they breastfed more frequently due to stay-at-home orders and delayed plans to wean their infant or young child. Key themes drawn from the qualitative data were: emerging knowledge and perceptions of the relationship between COVID-19 and breastfeeding, perceptions of immune factors in human milk, and the social construction of COVID-19 and infant and young child feeding perceptions and knowledge. There were immediate positive effects of stay-at-home policies on human milk feeding practices, even during a time of considerable uncertainty about the safety of breastfeeding and the transmissibility of SARS-CoV-2 via human milk, constrained access to health care services and COVID-19 testing, and no effective COVID-19 vaccines. Federally mandated paid postpartum and family leave are essential to achieving more equitable lactation outcomes.

## Introduction

Breastfeeding has been critical to postpartum and newborn health throughout the COVID-19 pandemic. Currently, all major health organizations, including the World Health Organization (WHO), United States Centers for Disease Control and Prevention (CDC), American Academy of Pediatrics (AAP), and the Academy of Breastfeeding Medicine (ABM) recommend breastfeeding along with vaccinations during pregnancy or lactation for optimal protection against SARS-CoV-2 infection and severe COVID-19 disease. However, these recommendations have evolved rapidly and have not always been implemented consistently within the United States or in other countries (Hoang et al., 2020; Tomori et al., 2020).

Early in 2020, as the first wave of the COVID-19 pandemic spread across the U.S., there was limited evidence regarding modes of disease transmission, pathogen history, and disease severity during gestation, parturition, and lactation. In this time of uncertainty, the WHO guidance recommended early breastfeeding initiation, immediate skin-to-skin care, and postpartum lactation support in hospital and community settings (WHO, 2020). However, in the U.S., many recommendations were in direct contradiction to the WHO, which led to the adoption of discordant COVID-19 related perinatal policies in medicine and public health (Hoang et al., 2020). For example, many hospital policies limited support persons during labor and delivery, enacted immediate postpartum separation of mothers with COVID-19 symptoms (Tomori et al., 2020) and limited or prohibited in-person skilled lactation support to postpartum patients in some hospitals (Perrine, 2020). These and similar policies have had significant detrimental effects on lactation outcomes, including timely initiation, exclusivity, and duration (Hoang et al., 2020).

Stay-at-home orders were enacted between March 1 and May 31, 2020, in 42 states and territories. This evidence-based practice significantly reduced movement and reduced community transmission (Moreland, 2020). It also meant that millions of infants and young children had more opportunities for close physical contact with their breastfeeding parent than they would have had were it not for the COVID-19 pandemic, in a country without federally protected paid postpartum/family leave policies.

The full impact of COVID-19 stay-at-home orders on breastfeeding, however, has not been fully appreciated. In low- and middle-income countries (LMIC), immediate postpartum maternal-infant separation policies, in particular, likely resulted in significant excess maternal and infant mortality and morbidity (Rollins et al., 2021). However, there is limited research on these disruptions vis-à-vis infant and young child feeding in high income countries (HIC), such as the U.S., particularly within sociology and anthropology.

The U.S. stands out among HIC, because it has no guaranteed paid leave during the postpartum period (International Labour Organization, 2017; Steurer, 2017). A lack of federally mandated paid postpartum and family leave in the U.S. is a major structural barrier to improving breastfeeding rates maternal and health outcomes across the nation (Griswold and Palmquist, 2018; Kim et al., 2019). This policy failure does not affect all U.S. populations in the same way and contributes to significant breastfeeding disparities that are stratified by race, ethnicity, income, type of employment, and education (CDC, 2022a).

Emergent infectious diseases introduce critical public health challenges related to how rapidly changing scientific information, guidance from health care organizations, and local epidemiological factors influence societal perceptions of disease transmission, interpretation of emerging scientific information, and human behaviors (Hahn and Inhorn, 2008; Farmer et al., 2013). Thus, the purpose of this paper is to describe breastfeeding intentions, practices, and experiences during the first wave of the COVID-19 pandemic in the U.S. We draw upon critical biocultural approaches in anthropological study of breastfeeding (Tomori et al., 2018) to examine the role of social institutions and policies in shaping breastfeeding practices, against the backdrop of the emergent COVID-19 pandemic in the U.S. This study offers insights into how some parents of infants and young children in the U.S. navigated uncertainty in feeding decisions against a backdrop of rapidly changing, and often contradictory guidance, related to COVID-19. Our study contributes to this special issue, and to the broader social science literature, by exploring the intersection of first wave COVID-19 policies and breastfeeding.

## Materials and methods

### Study design

A cross- sectional, mixed-methods study design (Bernard, 2018) was used to generate both quantitative and qualitative data regarding breastfeeding practices during the early onset of COVID-19 in the U.S. This approach was responsive to the urgent nature of data collection during this period of the COVID-19 pandemic (Johnson and Vindrola-Padros, 2017; Richardson et al., 2021). It also facilitated collection of a robust set of quantitative and qualitative data in a short time frame, within the constraints of social distancing. Structured survey items were included to gather detailed data on lactation outcomes and feeding practices. Open-ended survey questions were included to elicit descriptive responses, which enrich our understanding of the study findings and strengthen our interpretations of the quantitative data (Bernard, 2018; Esposito and Evans-Winters, 2021).

### Study population and recruitment

Data were collected among a convenience sample of respondents who completed an open access online survey March 25, 2020–May 31, 2020. Participants were recruited across several social media platforms, including Facebook and Twitter as well as through email listservs. No calculations for sample size adequacy were required for the study, as we were not testing specific hypotheses, and we were not aiming to recruit a population-based representative sample.

After confirming their informed consent to participate, survey respondents were presented with a set of eligibility screening questions. Individuals were eligible if they indicated they were at least 18 years of age, were currently feeding an infant or young child that was 2 years of age or younger, and residing in the United States. The survey was only available in English, and it required respondents to access it with a live internet connection and type in responses. No other exclusions were applied. Ethics approval for this study was granted after review by the Washington University in St. Louis Institutional Review Board #2020003141 (Quinn, PI).

### Survey design and data integrity

The online survey was accessible by both computers and phones, enabled to allow only one attempt per IP address, and contained adaptive design to reduce participant burden (Andrews et al., 2003). The survey was also disseminated following established guidelines to protect participant privacy, including using open-access distribution of the survey link, separation of consent from survey responses and any personally identifiable information, not including external links from third-party sites in the survey, conducting a privacy audit, data encryption, and clear disclosure of sampling procedures (Cho and Larose, 1999). Increasingly, software programs that imitate the behavior of a human by performing automated tasks on the Internet (i.e., “bots”) compromise the integrity of data collected using web-based survey instruments (Buchanan and Scofield, 2018). In light of these concerns, prior to preparing data for analyses we used a stepwise process to screen the surveys for bot interference and clean the data, using recommended indexes outlined by Dupuis et al. (2019): response coherence, response reliability, Mahalanobis distance, person-total correlation, psychometric antonyms and synonyms, odd-even consistency, and longest-string.

The survey elicited 2,400 unique responses. After screening for possible bot generated data and eliminating all compromised responses as described above, the final sample size was 1,437 responses. This response rate is in line with the typical ranges reported for actual and not bot generated survey responses (Pozzar et al., 2020; Griffin et al., 2021). Using adaptive skip logic likely promoted a lower percentage of bot-generated data (Pozzar et al., 2020). Average time spent answering the survey was 14 min after removing suspected bot data (one criteria for bot data was survey answer time of <4 min as it was estimated that given the number of survey questions a human could not respond to the short form of the survey in <5 min). Given the dissemination of the survey link, it was impossible to calculate the rate of individuals who clicked on the survey link but decided not to participate after reading the consent form or who started the survey but exited out before completing the survey; only 5 individuals clicked “decline to participate.” Thirty-one (1%) of individuals who consented to participate failed the eligibility screener and did not take the survey.

### Measures and analysis

Quantitative measures for the online survey included questions used to gather information with which to better characterize the convenience sample. It also contained items regarding infant and young child feeding practices, which are commonly included in national surveys (CDC, 2021; CDC-PRAMS, 2022) but were adapted to fit the specific context of COVID-19. Additional ethnographically informed survey questions were developed by the investigator team based upon previous anthropological research on breastfeeding and human lactation. These items facilitated deeper exploration of the nuances of infant and young child feeding, given the challenges of the early COVID-19 pandemic and related policies and practices.

Respondents were asked to answer 58 survey questions as follows: demographic questions (respondent age, age of youngest infant, parity, state of residence, race, ethnicity, estimated household income, employment status, type of work, marital status), 34 infant feeding questions (current feeding [5 items], prenatal feeding intentions [2 items], complementary feeding [1 item], breastfeeding frequency [2 items], formula feeding [10 items], changes in infant feeding [2 items], weaning [10 items], partner support [2 items]), and 14 questions related to self-reported COVID-19 testing, screening, and diagnosis. Ten items were open-ended questions including: perceptions of COVID-19 and breastfeeding, description of reasons for any changes in infant and young child feeding practices due to COVID-19, description of sources of information that respondents used to make feeding decisions, and responses related to feeding experiences.

Descriptive analyses of quantitative data were performed using STATA 13.0 (StataCorp, 2013). ANOVA or chi-square tests were done, when appropriate, to test for between group differences. Percentages were compared using two-tailed, two sample z-tests. To further explore whether hospital maternal-newborn separation policies, stay-at-home policies, and contradictory policies for breastfeeding accounted for differences in infant feeding decisions, we tested whether differences in infant feeding were different based on timing of delivery (born before or after March 13, 2021) among infants <6 months of age. Because regional variation in the prevalence of COVID-19 may have explained some differences in infant and young child feeding practices, we assessed feeding practices by geographic location, using divisions defined by the U.S. Census Bureau. Given the extensively documented racialized disparities of COVID-19 in the U.S., we examined differences in infant feeding practices by respondent race/ethnicity.

Responses to open-ended survey questions were analyzed using a conceptual content analysis approach (Bernard, 2018; Esposito and Evans-Winters, 2021). First, all survey questions that had open-ended responses were reviewed sequentially and a subset was selected for the present analysis. Next, based on this initial review, a coding framework was developed for each question's set of responses. The codes were derived inductively (Bernard, 2018; Esposito and Evans-Winters, 2021) and enabled the research team to group together responses that contained similar text strings or phrases having similar meanings. Shared responses are theoretically significant to biocultural research on breastfeeding, as they give insight to shared or sociocultural nature of belief systems, knowledge, attitudes, and practices (Bernard, 2018). The coding framework was then reviewed and refined by the investigator team, after which time the codes were applied to the data. This approach to the content analysis facilitated enumeration of responses per assigned code. Finally, a thematic analysis was applied to the coded data. The investigator team reviewed the coded responses, and from the words and meanings conveyed in the responses, developed a set of sub-themes that were grouped together under a set of key overarching themes. The research team reviewed, assessed, and came to consensus regarding the themes to minimize bias and refine interpretations of the data. For the final report, the total proportion of coded responses was calculated by dividing the total number of participants who provided a response to the item by the responses within each theme. Analysis of the open-ended survey responses enhances our ability to interpret the quantitative results.

## Results

### Results of the quantitative analysis

#### Characteristics of the study sample

Demographic characteristics of survey respondents, including race and ethnicity, household income, and employment status, are summarized in Table 1. Ninety-seven percent of respondents indicated that they had given birth to their infant who was 2 years of age or younger, while 3% did not provide specific information regarding their relationship to the child. The average age of respondents was 32.8 years (range 19–48, SD 4.45). The average age of the participants' youngest infant was 7.8 months (range 0–24 months; SD 6.68). Race and ethnicity were assessed using U.S. census categorizations. Most respondents (87%) selected “white” and “non-Hispanic or Latina” race and ethnicity, respectively. Across all other racial categories, 15% selected either American Indian or Alaska Native, Asian or Asian American, Black or African American, or Native Hawaiian or Pacific Islander, and 4% selected Hispanic or Latina for ethnicity. Thirty-eight percent of respondents were primiparous, while all other respondents were multiparous (range 2–8; 93% of the sample had 3 or less children). We did not find significant differences in responses to any of the survey items by race/ethnicity, but our sample was not adequately diverse or powered explore differences with precision.

**Table 1:** Demographic characteristics of 1,437 U.S. respondents, ages 18 and older, currently feeding their infant or young child who is 2 years of age or younger, March–May 2022.

**Category**	***N***	**%**
**Respondent age**		
18–20	3	0.2
21–25	73	5
26–30	359	23
31–35	581	40
36–40	306	21
41–50	58	4
No response	87	6
**Racial categories**		
American Indian or Alaska Native	5	0.4
Asian or Asian American	50	4
Black or African American	67	5
Native Hawaiian or Pacific Islander	2	0.1
White	1,203	84
Not reported	110	8
**Ethnicity categories**		
Hispanic or Latina	50	4
Non-Hispanic or Latina	1,327	92
Not reported	60	4
**2019 household income**		
<$25,000	48	3
$25,000–49,999	125	9
$50,000–$74,999	234	16
$75,000–$99,000	221	15
$100,000–$149,999	373	26
$150,000–$199,999	181	13
$200,000–$249,999	79	6
>$250,000	122	9
Not reported	54	4
**Marital status**		
Legally married to one person	1,260	88
Cohabitating with one person	91	6
Single parent	27	2
Divorced and co-parenting	3	0.2
Not reported	56	4
**Parity**		
1 child	542	38
> 1 child	848	59
Not reported	47	3
**Working status**		
Working at home (due to COVID-19 policy)	406	28
Stay-at-Home parent (before COVID-19)	377	26
Healthcare worker	227	16
On maternity/family leave	200	14
Has always worked from home	83	5
Currently working in an office	53	4
Full-time student	23	2
Not reported	68	5

Respondents were located in all nine regions of the U.S., with most concentrated in the West North Central, South Atlantic, East North Central (Table 2). The fewest respondents were in the Mountain, West South Central, and New England regions. We found no statistically significant differences in infant and young child feeding by geographic region.

**Table 2:** Geographic distribution of infant and young child feeding practices among 1,381 survey respondents ages 18 years and older by the nine U.S. census division regions.

	**United States census division regions**									
	**1**	**2**	**3**	**4**	**5**	**6**	**7**	**8**	**9**	
	**New England**	**Mid-Atlantic**	**East North Central**	**West North Central**	**South Atlantic**	**East South Central**	**West South Central**	**Mountain**	**Pacific**	**Total**
Infant feeding category^*^										
Human milk	25	55	90	113	103	30	32	24	65	537
Human milk and formula	4	11	17	11	22	6	7	5	16	99
Human milk and solids	28	52	110	154	143	40	22	29	66	644
Human milk, formula, and solids	5	5	15	17	14	3	3	3	8	73
Formula only	0	1	1	6	1	0	1	1	0	11
Formula and solids	1	1	4	4	2	2	0	0	3	17
**Total**	63	125	237	305	285	81	65	62	158	**1,381**

* Human milk feeding includes breastfeeding, feeding with expressed human milk, or a combination of breastfeeding and feeding with expressed human milk. Solids includes any complementary foods in addition to human milk or formula.

#### Infant feeding intentions

A majority of all respondents (97%) indicated that when they were pregnant, they had an intention to exclusively feed their infant with human milk, either through breastfeeding, feeding with their own expressed milk, or some combination of these two strategies (Table 3). Only 5 participants indicated that when they were pregnant, they had intended to exclusively feed their infant with formula.

**Table 3:** Infant and young child feeding practices among survey respondents, ages 18 years and older, stratified by their child's age, March–May 2020.

**Survey item**	**All respondents**	**Stratified by child's age**	
		**0–5 m**	**≥6 m**
	***N %***	***N %***	***N %***
**Prenatal infant feeding intentions (0–6 months)**	**1,356**	**686**	**670**
Exclusive human milk feeding (breastfeeding, feeding expressed human milk)	98	94	97
Combination of human milk feeding and formula feeding	4	5	3
Exclusive formula feeding	0.4	0.4	0.3
**Current infant or young child feeding practice**	**1,397**	**688**	**703**
Exclusive human milk feeding	39	7	6
Human milk and formula feeding (no other solids or liquids)	7	13	2
Human milk, solids, and other liquids (no formula)	43	3	82
Human milk, solids, other liquids, and formula	9	10	8
Formula, solids, and other liquids (no human milk)	1	0.7	2
Exclusive formula feeding	0.8	1.6	0.1
**Change from prenatal intention to current feeding practice**	**193**	**116**	**77**
Change from exclusive human milk feeding intention to any formula feeding	92	87	99
Change from exclusive formula feeding intention to any human milk feeding	0	0	0
Change from combination feeding to exclusive human milk feeding	17	11	0
Change from combination feeding to exclusive formula feeding	2	2	1
**Change in plans to breastfeed child due to COVID-19 concerns**	**1,334**	**658**	**676**
Yes	15	11	19
No	85	89	81
**Self-efficacy in meeting intended human milk feeding goal**	**1,359**	**673**	**686**
Yes	74	69	81
No	6	10	3
Not yet	18	21	16
**Current mode of feeding human milk**	**1,338**	**661**	**677**
Donor milk only	0.5	0.5	0.4
Breastfeeding only	55	48	62
Breastfeeding and feeding my own expressed milk	35	40	31
Breastfeeding and/or feeding my own expressed milk and with donor milk	0.9	0.9	0.9
Exclusively feeding with my own expressed milk	8	10	6
**Changes to weaning plans due to COVID-19**	**381**	**172**	**209**
Earlier than planned	5	6	3
Later than planned	95	94	97

#### Breastfeeding frequency, milk expression, and milk storage practices

Most participants (66%) who were breastfeeding their infant prior to national implementation of stay-at-home orders indicated no change in their frequency of breastfeeding after March 13, 2020. However, 31% of respondents who reported breastfeeding their infants at least some of the time did report an increase in breastfeeding frequency, due to being at home with their infant more. Just 4% reported decreased breastfeeding frequency. Among the 1,352 respondents who indicated they were providing any human milk for their infant, 92% reported storing expressed milk for later use, unrelated to COVID-19. Although we did not identify significant differences in infant and young child feeding practices by race/ethnicity, when we explored responses to the question about changes in breastfeeding frequency, among all respondents of color, a higher proportion (8%) reported a decrease in breastfeeding due to stress as compared with 3% among only non-Hispanic/Latina white respondents.

#### Formula feeding

Exclusive formula feeding was the least common feeding practice. Combination feeding with both human milk and infant formula was relatively more common. Of the 688 respondents who provided information on complementary feeding, less than one percent reported early introduction of solids 0–5 months, and exclusive human milk feeding or formula feeding of infants >6 months was reported by 6% of the 703 respondents who answered this question. Among 256 respondents who were feeding their infant with any formula, 12% indicated they had recently purchased extra formula. Sixty-five percent of this subset indicated they were concerned about getting enough formula, and 71% indicated they had a fear of formula shortages. One respondent indicated that they received their formula through WIC.

#### Changes in feeding or weaning plans related to COVID-19

Respondents provided information on whether COVID-19 had led to any planned changes in when to stop breastfeeding or pumping and giving their infant expressed milk (Table 3). Of the 1,380 respondents who indicated an intention to exclusively feed their infant with human milk for the first 6 months, 75% reported meeting their goals and only 6% reported not meeting their intended goal. The remaining 18%, indicated not yet meeting their goal, likely because their infants had not yet reached 6 months of age or the age when they had planned to wean. Survey respondents who selected Black race and Hispanic/Latina ethnicity were less likely to report meeting their feeding goal than non-Hispanic/Latina white participants (8%, 14%, respectively vs. 6%). Household income was not associated with meeting respondents' infant feeding goals.

At the time of the survey, no participants reported infection with COVID-19 as a reason for not meeting their feeding intentions. Over two thirds (68%) indicated that they had no intention of altering their planned weaning age. However, 31% planned to delay weaning until the pandemic was over. Only 17 participants (1%) indicated plans to wean earlier, and fewer than one percent noted that they had already weaned but were attempting to relactate.

#### Spouse or partner support for feeding decisions during COVID-19

Of the 1,338 participants who reported that they had a spouse/partner, 99% indicated that their spouse/partner was supportive of their infant feeding decisions. Respondents were asked to provide information about partner/spousal preference for timing of weaning. Of the 1,335 participants who provided this information, 95% indicated no preference, 5% reported a preference for weaning later than planned, and only 0.4% indicated that a spouse/partner expressed a preference for weaning earlier than planned.

#### Differences in feeding based on timing of birth

Given how early the survey was launched during the pandemic, only 9% of participants reported giving birth during the pandemic, with the other 91% having given birth prior to March 13, 2020, as this is the date of the Proclamation on Declaring a National Emergency Concerning the Novel Coronavirus Disease (COVID-19) in the U.S. To compare the impacts of giving birth during the initial stage of the pandemic, we compared this subset to the 440 individuals who had given birth prior to March 13, 2020 (Table 4). There were no significant differences between the two groups by type of infant feeding.

**Table 4:** Comparison of infant feeding at the time of survey among 574 respondents with infants <6 months of age, stratified by a delivery date before or after March 13, 2020.

	**Infant**<**6 mos, born before March 13, 2020**		**Infant**<**6 mos, born after March 13, 2020**		**Total**
Infant feeding category^*^	**Number**	**%**	**Number**	**%**	
Human milk	355	81	106	79	461
Human milk and formula	55	13	22	16	77
Human milk and solids	20	5	2	1	22
Human milk, formula, solids	1	0.2	0	0	1
Formula only	6	1	4	3	10
Formula and solids	3	0.7	0	0	3
**Total**	**440**		**134**		

* Human milk feeding includes breastfeeding, feeding with expressed human milk, or a combination of breastfeeding and feeding with expressed human milk.

Solids includes any complementary foods in addition to human milk or formula.

#### Suspected or confirmed COVID-19 and infant feeding decisions

At the time of the survey, access to COVID-19 testing was comparatively more difficult than later in the pandemic. As such, we measured COVID-19 incidence in two ways: laboratory test confirmed SARS-CoV-19 and suspected COVID-19 infection based on symptoms. Three percent of 1,390 respondents reported a confirmed COVID-19 test at the time of survey. Of the 33 respondents with confirmed positive COVID-19 tests, 66% continued directly breastfeeding their infant; 15% were feeding exclusively with expressed human milk, and 10% had stopped breastfeeding or feeding with expressed milk. Among those with symptoms, 72% continued directly breastfeeding, 14% wore masks while breastfeeding, 8% switched from breastfeeding to exclusively pumping, and 2% stopped breastfeeding altogether. The remaining participants were either not feeding human milk or did not respond to the question. Among 1,377 participants who provided information about whether anyone within their social network had been diagnosed with COVID-19, 70% did not know anyone with COVID-19, 30% knew someone with COVID-19, and 0.2% reported COVID-19 among someone in their household.

## Results of the qualitative analysis

A summary of participants' open-ended responses is presented in Table 5. Illustrative verbatim quotes from the survey responses are included. The responses are organized by key themes, which complement the quantitative survey results presented above.

**Table 5:** Themes, sub-themes, and illustrative quotes from survey respondents, ages 18 years, and older currently feeding an infant or young child 2 years of age or younger, March–May 2020.

**Theme**	**Sub-theme**	***N***	**%**	**Illustrative quotes**
Emerging knowledge and perceptions of the relationship between COVID-19 and breastfeeding, human milk	*No information*	332	23	“Nothing. But typically you're told to continue nursing while sick.”
				“Nothing - early weaning will be due to low supply from stress”
				“Nothing specifically but I know breast milk can help keep my child healthy”
	*Uncertain transmission*	248	17	“Unlikely passed through breast milk, but they don't know for sure”
				“COVID-19 can't be transmitted via breastmilk, unknown if antibodies are transmitted”
				“That it is not believed to be passed to infants in milk, but that little is known”
	*Safe/Fine*	127	9	“That it is safe to continue breastfeeding if I have COVID-19, just to take precautions to protect baby (wash hands, wear mask).”
				“that it is safe, even for moms with a COVID-19 diagnosis like me”
				“WHO said it is safe to continue nursing even with symptoms or a positive test”
	*Pump if you have COVID*	32	2	“I have heard that it is still recommended to feed your infant breastmilk, even if you have the virus.”
				“It's recommended to pump and feed, not at breast”
				“That it is safe to pump and bottle feed but not to nurse directly if you test positive”
	*Stop breastfeeding*	21	1	“That you should not breast feed if you are infected”
				“To stop nursing if you have it”
				“That the cdc recommends not breastfeeding and separating mother and child if mom tests positive but the WHO says to continue breastfeeding”
Perceptions of immune factors in human milk and COVID-19	*Keep breastfeeding*	291	20	“I have heard that breastmilk is the best medicine for baby if mom is positive for COVID-19 and to keep breastfeeding (with extra sanitary precautions) if possible.”
				“I've read the recommendation is it's ok to continue breastfeeding if you have COVID-19 but same article also said there's been no studies done to confirm or dispute recommendation.”
				“Breastmilk has super hero powers and to keep going if you test positive”
	*Antibodies*	192	13	“That like with other viruses breastmilk can convey antibodies to fight the virus. And that my baby would likely get the virus if i had it whether i breastfed or not.”
				“That you should continue to breast feed and if you do have it to continue to feed, baby may get antibodies that protect them against COVID-19”
				“Breast milk likely contains antibodies to help fight off COVID-19, if mother has been infected.”
	*Immune benefits*	144	10	“The best way to protect my child is by continuing to provide her with breast milk.”
				“Breastmilk contains antibodies to help fight off any infection”
				“Most effective at fighting illness/decreasing severity...the best protection”
	*Delay weaning*	40	3	“Keep nursing/pumping and delay weaning”
				“Breastmilk has antibodies (already knew) and so it's recommended to delay weaning currently if possible specifically for COVID-19 protection”
				“Best to continue nursing or pumping and delay weaning. If mother is presenting symptoms, wear a mask while feeding and wash hands before and after touching baby.”
Social construction of COVID-19 and infant and young child feeding perceptions and knowledge	*Social influences*	22	10	“One Facebook post that said wear a mask”
				“I have seen many articles on all sides including not breastfeeding if you have COVID-19. I am following WHO recommendations”
				“I've heard some ladies in forums say if their partners get it they will all be drinking breast milk. I have also been advised to continue breastfeeding if I get it.”
				“That some hospitals are separating moms and newborns. This should NOT happen. Babies need breastmilk for the antibodies to fight COVID-19 especially if Mom has symptoms.”

### Perceptions and knowledge of COVID-19, breastfeeding, and transmission through human milk

Under the theme of knowledge and perceptions of COVID-19 transmission via breastfeeding and human milk were a wide range of responses to two open-ended questions: “What have you heard about breastfeeding and COVID-19?,” and, “What have you heard about breastmilk and COVID-19?” Responses that were coded as a “no information” were the most common. Yet, even when respondents indicate they had heard “no information,” they typically offered a brief description of ways that they had filled the knowledge gaps. For example, one person noted that:

*I have read a lot about the potentially protective qualities of breast milk, along with the emotional/attachment benefits for both parent and child during this stressful time*.

Another respondent shared:

*I've heard that vertical transmission is unlikely. But I also know that there's a lot we still don't understand about the virus. If I show any symptoms, I'll be in close touch with pediatrician, as well as my physician*.

Responses coded under the sub-theme “Uncertain transmission,” were similar in that they used terms or phrases such as *unlikely, not for certain, unknown*, or *not for sure*, to describe a low perceived likelihood that COVID-19 would be transmitted via breastfeeding or human milk. Other responses were aligned with the available science and guidance at the time:

*There is potential for exposure when feeding at the breast. Little is known about conferring immunity through breast milk*.

Fewer responses were coded under the sub-theme “Safe/Fine.” These responses were similar to those coded as “Uncertain transmission,” but with a stronger degree of alignment with WHO guidance. The final two sub-themes, “Pump if you have COVID” and “Stop breastfeeding” had markedly fewer total responses, respectively, than the others. The former reflected information that if a breastfeeding parent had COVID-19, it was safer to pump than to breastfeed. The latter included a range of responses, which mirrored CDC guidance and many hospital polices at the time, that breastfeeding or feeding the baby with expressed milk should be stopped if a mother had COVID-19.

### Perceptions of immune factors in human milk and timing of weaning

The second key theme was based on responses that specifically reflected “perceptions of immune factors in human milk and COVID-19.” Responses that mentioned some aspect of the relationship between human milk and immune properties were found in responses across various open-ended survey questions. Three sub-themes were developed to capture nuances in the range of responses under this key theme (Table 5).

“Keep breastfeeding” is a sub-theme that connects perceptions of the immune properties of human milk to recommendations, or the rationale for, decisions to continue breastfeeding in the context of suspected or confirmed COVID-19, for example:

*The antibodies in breast milk may be beneficial to infants who could be exposed to covid-19*

Across these responses, participants discursively link breastfeeding or providing expressed milk to their infant as a positive action they can take during the uncertainty and stress of the pandemic:

*I want to continue giving my baby as many good benefits of breast milk as possible during this stressful and uncertain time*.

The term *antibodies* appeared in 192 responses. Antibodies in these responses are generally perceived as the specific mechanism in human milk that *fights off* COVID-19 infection, especially in the absence of vaccines or known treatments, for instance:

*Breastmilk provides special nutrients and antibodies to my child so I want to continue offering that to my baby in case it gives him any extra protection against COVID19*.

Similarly, words and phrases that speak to the relationship between breastfeeding and human milk with immunological responses to infection such as *resistance, protection*, and *decreasing severity* underlie respondents' decisions to continue breastfeeding, sometimes longer than intended due to the pandemic, even when it was not well-established whether breastfeeding protected infants from severe COVID-19 or if the SARS-CoV-2 virus could be transmitted to an infant via breastfeeding or expressed milk. These perceptions of the immunological importance of human milk were the impetus for many respondents indicating that they had changed their decision about the timing of weaning because of the COVID-19 pandemic. Continued breastfeeding was a decision that some made to do whatever they could to help prevent their infant from becoming sick:

*I'm worried about the baby getting sick and want him to have breast milk longer than 6 months*

*I want the best for my child and it's scary to think about children getting sick. Breastfeeding seems to be a logical way to support his health*.

The decision to delay weaning introduced challenges for some respondents. For example, the continuation of breastfeeding was viewed as disrupting one respondent's transition to having greater bodily autonomy in order to protect their child:

*I'm torn. I want my body back to myself but I need her to be safe as well*.

Another respondent described attempting to continue breastfeeding despite a desire to wean and the difficulties of trying to continue making milk while stressed during the national stay-at-home orders, when children could not yet return to school:

*I'm ready to be done breastfeeding and am stressed now that none of my kids are in school (lower supply), but I'm trying to make it a little longer*.

A number of respondents, though, noted that staying at home was a welcome opportunity to continue breastfeeding or pumping and bonding with their baby, an opportunity that would not be possible except for the stay-at-home orders. In response to the question “How do you feel about your decision to delay weaning,” one respondent replied:

*I feel good, because I'm already in the routine of feeding and pumping. Plus I want to protect my child. I have always been nervous about weaning and engorgement, but am happy to get to continue to bond with my child in that way*.

Others were ambivalent about the tensions between delayed weaning and the well-being of their infant:

*Not good. Guilty for my own stress affecting her*.

Delayed weaning exacerbated worry for some who wanted to continue breastfeeding, but were concerned about meeting their goals and also exposing their baby to COVID-19:

*I am worried that I won't be able to meet my goals or that I am being selfish and will expose the baby while direct breastfeeding*.

### Social influences on perceptions of COVID-19 transmission via human milk

A final key theme was developed to convey the range of responses that illustrated the social construction of human milk and breastfeeding safety during the early COVID-19 pandemic. The sub-theme “Social influences” includes responses to the various sources of information on COVID-19 and breastfeeding and/or expressed human milk feeding. These responses included social networks, social media and online communities, word-of-mouth from friends and family, and anecdotal stories and experiences. These responses illustrate how respondents turned to sociocultural influences as a way to make sense of often ambiguous or conflicting information about COVID-19 and then translated it into various decisions about their own infant feeding practices.

## Discussion

The COVID-19 pandemic has introduced unprecedented challenges for people experiencing pregnancy and postpartum and for parents who are caring for their infants and young children. Our study contributes to a growing literature on how COVID-19 mitigation policies have affected infant and young child feeding practices in the U.S. (Palmquist et al., 2020; Pasadino et al., 2020; Patil et al., 2020; Perrine, 2020; Snyder and Worlton, 2020; Bartick et al., 2021; DeYoung and Mangum, 2021; Gutschow and Davis-Floyd, 2021; Mollard and Wittmaack, 2021; Schindler-Ruwisch and Phillips, 2021; Cohen and Botz, 2022), while offering a unique perspective from the earliest phase of the pandemic.

During the early phase of the COVID-19 pandemic, rapidly changing guidance about maternity care practices in the immediate postpartum period due to suspected or confirmed COVID-19 raised serious concerns about the potential impacts of maternal-newborn separation policies on breastfeeding outcomes (Tomori et al., 2020). During the same period, stay-at-home orders were also imposed, leading to dramatic changes in daily life for millions in the US. While there has been critical analyses of maternity care policies and their consequences for breastfeeding in both high-income (Thomson, 2022; Turner et al., 2022) and low-income countries (Rollins et al., 2021), the full impact of pandemic policies on lactation have not been fully investigated.

In our study, strong breastfeeding intentions during pregnancy appear to be related to a high prevalence of breastfeeding and feeding with expressed human milk during the onset of the COVID-19 pandemic. Nearly all the survey respondents indicated a strong intention to breastfeed or feed their infant with expressed human milk exclusively 0–6 months. Perceptions, worries, and concerns regarding COVID-19 transmission were less apparent among respondents who indicated a strong prenatal intention to breastfeed. Similarly, for two thirds of study participants, breastfeeding frequency and the planned timing of weaning did not change because of COVID-19. In fact, nearly a third of participants delayed weaning. Additionally, findings from the present study point to an increased frequency of breastfeeding, delayed weaning, a strong belief in the role of human milk in protecting infants, and being able to stay-at-home during the postpartum year, as a multi-dimensional buffer against the negative effects of COVID-19 hospital separation policies on lactation.

Qualitative findings illustrate that most respondents were at least partly motivated to continue breastfeeding and delay weaning, due to knowledge and perceptions of human milk as providing immunological protection to their infant, even when there was not yet sufficient data to confirm this. Immune factors in human milk are a cornerstone of U.S. medical and public health education to promote breastfeeding (CDC, 2022b). The immunological factors in human milk have been shown elsewhere to be critical to parents' decisions to breastfeed, to donate milk to milk banks and milk sharing communities, and to accept donor milk and shared milk when available.

In an ethnographic study of community milk sharing in the U.S., the idea of a “super immune system” was salient among mothers feeding their infants with milk from multiple donors (Palmquist, 2018). Similarly, in the present study, the idea of milk “having super powers” is significant to theorizing lactation in medical anthropology.

We now have substantial evidence that human milk is indeed critical to conferring passive immunity to infants when their breastfeeding parent has experienced SARS-CoV-2 infection or when their breastfeeding parent has been vaccinated (Perez et al., 2022; Whited and Cervantes, 2022; Young et al., 2022). Familiarity with the importance of human milk to infants' immune response, combined with strong breastfeeding intentions and economic security were also significant in respondents' decisions to continue breastfeeding and delay weaning during the first wave of COVID-19 in the U.S. This points to the continued importance of emphasizing the protective effects of breastfeeding against infectious disease in public health messaging, incorporating lactation in public health emergency planning, and providing structural support for realizing breastfeeding intentions, especially during emergencies.

Relatively few respondents indicated that they had weaned earlier than planned or would stop breastfeeding if they were diagnosed with COVID-19. At the same time, participants who reported that they would, indeed, stop breastfeeding if diagnosed with COVID-19 noted that they had heard that this was their pediatrician's policy or a public health recommendation they had heard. Only a small number of study participants indicated they were diagnosed with COVID-19. At the time of the survey community public health COVID-19 testing centers and at-home testing kits were not widely available or accessible, which may have contributed to underdiagnosis of infection. More respondents who reported a confirmed COVID-19 diagnosis stopped breastfeeding or feeding with expressed milk than those who reported continuing to breastfeed or offering their infant expressed milk. Some participants may have been discouraged from breastfeeding by CDC recommendations and hospital policies that undermined breastfeeding.

Only 13% of our study respondents gave birth during the pandemic, therefore, our findings are skewed toward those who experienced the onset of the pandemic after they already initiated their infant feeding journey, with most having already established lactation at the time of taking the survey. However, even among respondents whose infants were born after stay-at-home orders were implemented nationally, breastfeeding initiation and exclusivity 0–6 months was higher than the typical national averages prior to COVID-19 (CDC, 2022a). The study sample was also comprised of a majority of racially privileged, well-educated, partnered, and employed respondents. These characteristics are commonly associated with populations that have more resources and power to overcome structural barriers in breastfeeding initiation in the U.S. (CDC, 2022a).

Our study sample had much more positive breastfeeding experiences than more representative studies, especially among those who gave birth after the declaration of the pandemic in the U.S. when maternity care policies were significantly disrupted. For example, a survey of 1,344 U.S. hospitals between July 15 and August 20, 2020 revealed that while a majority of hospitals reported no significant change in breastfeeding initiation rates, COVID-19 impacted postpartum lactation support for mothers with suspected or confirmed COVID-19 (Perrine, 2020). Moreover, the report identified a number of hospitals that either prohibited or discouraged skin-to-skin contact, direct breastfeeding, and milk expression when COVID-19 was suspected or confirmed. The report also noted the trend to accelerate hospital discharge to <48 h after birth among a majority of hospitals, suggesting a critical need for postpartum follow-up and remote monitoring (Perrine, 2020). Other studies using longitudinal cohort data showed that even when mother-infant dyads initiated breastfeeding in the hospital during the COVID-19 pandemic, they were less likely to have continued breastfeeding after discharge (Popofsky et al., 2020; Bartick et al., 2021; Mollard and Wittmaack, 2021). Similarly, a study examining California WIC data compared breastfeeding outcomes of infants prior to and after March 2020 in Los Angeles County (Koleilat et al., 2022) concluded that, among this low-income population, breastfeeding initiation and any breastfeeding at 3 and 6 months decreased significantly among infants born during the COVID-19 pandemic. Finally, recent work demonstrated that direct breastfeeding in hospital was associated with 5.7 times greater likelihood of any human milk feeding and 8 times greater odds of direct breastfeeding post discharge (Rostomian et al., 2022), demonstrating the detrimental impacts of early separation policies.

Although we did not find significant differences in infant feeding based on geography, hospitals in communities with a high burden of COVID-19 infection early in the pandemic reported more significant declines in breastfeeding initiation rates in hospital and after returning home, suggesting some geographic variability in how separation policies affected lactation support services (Popofsky et al., 2020). Qualitative and mixed-methods studies have shown that perinatal mental health and psychosocial support, access to remote/telehealth health care services, and skilled lactation support were all important to buffering against COVID-19 separation policies and supporting breastfeeding during this time (Snyder and Worlton, 2020; DeYoung and Mangum, 2021; Schindler-Ruwisch and Phillips, 2021; Cohen and Botz, 2022). These findings warrant further inquiry into the ways that different populations parse conflicts between their own lived experience, perceptions of risk, social constructions of risk, and biomedical or public health authority in the wake of emergent infectious diseases and other emergencies. These emergencies are predicted to accelerate driven by climate change (IPCC, 2022), therefore, infant and young child feeding in emergencies (IYCF-E) policy planning and implementation are urgently needed.

Previous research has demonstrated both positive and negative impacts of the stay-at-home orders on perinatal and postpartum outcomes and experiences (Davis-Floyd et al., 2020; Farewell et al., 2020; Gildner and Thayer, 2020; Hynan, 2020; Premkumar et al., 2020; Kotlar et al., 2021). In our study we only explored stay-at-home orders in relation to COVID-19 and infant and young child feeding goals, practices, and experiences. Stay-at-home orders enabled a third of parents in our study to spend more time with their infants, which gave them an opportunity to feed their babies more frequently and to breastfeed for longer durations than would have been possible otherwise. There were immediate positive effects of stay-at-home policies on breastfeeding and human milk feeding practices, even during a time when there was considerable uncertainty about the safety of breastfeeding and the transmissibility of SARS-CoV-2 in human milk, constrained access to health care services and COVID-19 testing, and no effective COVID-19 vaccines (Hoang et al., 2020; Tomori et al., 2020).

There is a strong relationship between paid leave and the ability to realize breastfeeding desires and achieving more equitable breastfeeding outcomes (Vilar-Compte et al., 2022). Studies examining the impact of enacting paid parental leave on breastfeeding in California and New Jersey demonstrated that these state-level policies were associated with increased rates of both breastfeeding and exclusive breastfeeding (Huang and Yang, 2015; Hamad et al., 2019). After California introduced the first paid family leave program in the U.S., exclusive breastfeeding increased by 3–5 percentage points for the first three and 6 months, and then 10–20 percentage points for breastfeeding through three, six, and nine months (Huang and Yang, 2015). Comparing California and New Jersey, two states that implemented paid family leave policies, with the rest of the United States, Hamad et al. (2019) found that these policies were associated with a 1.3 percentage point increase in exclusive breastfeeding at 6 months. Notably, these benefits were more pronounced for higher-income, married, and white parents (Huang and Yang, 2015; Hamad et al., 2019). In contrast, we know from other research that childbearing people in communities of color are most negatively affected by structural racism in the U.S. and have fewer opportunities to leverage political economic, social, and legal resources that might buffer against the harmful effects of extreme economic pressures that force a return to work soon after giving birth (Ehrenreich and Siebrase, 2014; Hawkins et al., 2015; Johnson et al., 2015; Asiodu et al., 2021; Butler et al., 2021; Hemingway et al., 2021; Morrow et al., 2021). COVID-19 has only exacerbated these longstanding structural inequities that contribute to ongoing breastfeeding disparities in the U.S.

Our study participants, who were predominately economically secure, non-Hispanic/Latina white postpartum parents were able to sustain their breastfeeding intentions, initiation, or continuation. Across all levels of household income, white respondents indicated they were able to breastfeed or feed their infants with expressed milk as they had intended. Juxtaposed against reports of the disproportionate burden of COVID-19 related perinatal morbidity and mortality among minoritized and marginalized racial/ethnic groups in the U.S. (Woodworth, 2020), our study illustrates that breastfeeding as intended is another way that racial privilege was experienced during COVID-19 pandemic (Bassett et al., 2020; Gravlee, 2020; Poteat et al., 2020; APM Research Lab, 2021).

### Strengths and limitations

Our study has a number of strengths and limitations. It is one of the few studies to report on the early phase of the pandemic, thereby providing a unique window into a specific timepoint of the early phase of the pandemic with significant uncertainty, rapidly changing guidance, and large-scale mitigation policies.

It is well-established that web-based surveys that collect self-reported data have both discrete advantages and disadvantages. One advantage is that online surveys are useful for accessing specific interest groups that may not be widespread within the general population (Wright, 2005). Web-based surveys also bias participant selection, as they automatically require access to the internet, and in this case both digital literacy and English literacy, in order to complete the survey (Couper, 2000). Web-based surveys may not be representative of the general population and the widespread sharing of links makes it impossible to calculate refusal rates (Andrews et al., 2003). Self-reported data and open-ended responses to online surveys must be taken at face-value, since “validating” responses is often not possible or relevant (Bernard, 2018).

A strength of the study is that the convenience sample included responses from participants in every U.S. state and the District of Columbia. However, the size of the convenience sample and its lack of demographic diversity made it difficult to detect any differences in outcomes by race, ethnicity, income, employment or other social determinants of health through statistical analyses. Rapid response population-based research with a diverse sample of respondents is needed to better understand how pandemics disrupt breastfeeding practices as well as to understand how best to intervene. The timing of the survey is both a strength and a weakness. On the one hand the survey provides insight to breastfeeding transitions during a pivotal moment during the COVID-19 pandemic in the U.S. On the other, most respondents had given birth prior to COVID-19 stay-at-home orders, which limits our ability to interpret how hospital separation policies may have impacted breastfeeding outcomes.

Our mixed-methods anthropological study was developed in rapid response to the onset of the COVID-19 pandemic. Due to the nature of this rapid response, neither the survey nor the recruitment strategies were developed in collaboration with research stakeholders. A collaborative and participatory approach to designing and disseminating the survey would have strengthened recruitment of a more diverse participant population.

Another strength of our study was our ability to recruit a large number of participants. However, we recognize that people who agree to complete online research surveys without remuneration, have higher education attainment, higher incomes, and are disproportionally white (Witte et al., 2000). The COVID-19 pandemic introduced significant disruption to health care access, employment, housing, food, and job security for expectant and new parents across the nation. Study participants are biased toward individuals with not only the motivation, digital literacy, and English language fluency to complete this survey, but also the time and economic resources to do so without receiving a stipend. Study findings cannot be used to generalize to the U.S. population.

## Conclusion

Breastfeeding has been integral to supporting perinatal and newborn health outcomes throughout the COVID-19 pandemic. Federally mandated paid postpartum and family leave is essential to achieving more equitable lactation outcomes, and ultimately, improved maternal, child, and family health, population health, and economic prosperity (Rollins et al., 2016; Tomori et al., 2022). COVID-19 stay-at-home orders provided a natural experiment in which to document the potential impacts of paid leave for a socioeconomically diverse breastfeeding population in the U.S.

Around March 2020, it was unclear how long stay-at-home policies would be required and what the trajectory of the pandemic would be for the U.S. In September 2022, at the of writing this publication, COVID-19 continues to significantly impact the U.S. Since 2020 we have experienced multiple waves of critical rises in infection rates, emergence of variants, sustained high levels of community transmission, and over 1,051,389 deaths (National Center for Health Statistics (NCHS), 2022). Ongoing research is needed to understand the biocultural dimensions of breastfeeding as an adaptation to public health emergencies, including COVID-19. There is an urgent need to document how to respond to public health emergencies in ways that allow all communities to overcome structural barriers to breastfeeding and reduce breastfeeding inequities. In addition to supporting the basic human rights of all childbearing people, in the U.S., federally mandated policy for postpartum leave would greatly strengthen lactation outcomes, breastfeeding equity, and all associated maternal and infant health indicators across the life course and generations.

## Data availability statement

The datasets presented in this article are not readily available because we do not have permission to share the data. Requests to access the datasets should be directed to equinn@wustl.edu.

## Ethics statement

The studies involving human participants were reviewed and approved by Institutional Review Board, Washington University in St. Louis. The patients/participants provided their written informed consent to participate in this study.

## Author contributions

AP, EQ, and CT designed the study. EQ, CF, and SC prepared the data for analysis and conducted the analyses. AP, EQ, CT, and KT verified the analytic methods. AP took the lead in writing the manuscript. All authors provided critical feedback and helped shape the research, analysis, and finalizing the manuscript.

## Conflict of interest

The authors declare that the research was conducted in the absence of any commercial or financial relationships that could be construed as a potential conflict of interest.

## Publisher's note

All claims expressed in this article are solely those of the authors and do not necessarily represent those of their affiliated organizations, or those of the publisher, the editors and the reviewers. Any product that may be evaluated in this article, or claim that may be made by its manufacturer, is not guaranteed or endorsed by the publisher.
